# A novel HSP90 inhibitor, 17-DMAG, induces Tax down-regulation and its oral administration to ATL-model mice intervenes against the infiltration property of the ATL-like lymphocytes and provides extended survival period

**DOI:** 10.1186/1742-4690-11-S1-P44

**Published:** 2014-01-07

**Authors:** Emi Ikebe, Akira Kawaguchi, Kenta Tezuka, Shinya Taguchi, Satoshi Hirose, Takashi Matsumoto, Takahiro Mitsui, Kazuyo Senba, Akira Nishizono, Mitsuo Hori, Hiroo Hasegawa, Yasuaki Yamada, Takaharu Ueno, Yuetsu Tanaka, Hirofumi Sawa, William Hall, Yasuaki Minami, Kuan-Teh Jeang, Masao Ogata, Kazuhiro Morishita, Hideki Hasegawa, Jun-ichi Fujisawa, Hidekatsu Iha

**Affiliations:** 1 Department of Infectious Diseases, Oita University, Faculty of Medicine, Japan; 2 Department of Pathology, National Institute of Infectious Diseases, Japan; 3 Department of Microbiology, Kansai Medical University, Japan; 4 Department of Hematology, Ibaragi Pref. Central Hospital, Japan; 5 Department of Laboratory Medicine, Nagasaki University Graduate School of Biomedical Sciences, Japan; 6 Department of Immunology., Graduate School of Medicine University of Ryukyus, Japan; 7 Department of Molecular Pathobiology, Hokkaido University Research Center Zoonosis Control, Japan; 8 Department of Medicine and Microbiology, Centre Research Infectious Disease, Conway Institute Biomolecular Biomedical Research University, Coll. Dublin, Ireland; 9 Department of Biotechnology, Maebashi Institute Technology, Japan; 10 Laboratory and Molecular Microbiology, National Institutes of Health, USA; 11 Department of Hematology, Oita University, Faculty of Medicine, Japan; 12 Department of Medical Sciences, Faculty of Medicine, University of Miyazaki, Japan

## 

In the peripheral blood leukocytes (PBL) infected with human T-cell leukemia virus type-1 (HTLV-1), which causes HTLV-1 associated diseases including adult T-cell leukemia (ATL), HTLV-1 associated myelopathy (HAM) and HTLV-1 uveitis (HU), NF-κB-mediated anti-apoptotic signals or inflammatory signals are constitutively activated primarily by the HTLV-1 encoded oncoprotein Tax.

Tax interacts with the I-κB kinase regulatory subunit, NEMO, to activate NF-κB, and this interaction is maintained in part by a molecular chaperone, Hsp90, and its co-chaperone Cdc37. The antibiotic geldanamycin (GA) inhibits Hsp90’s ATP binding for its proper interaction with client proteins. Administration of a novel water soluble and less toxic GA derivative, 17-dimethylaminoethylamino-17-demethoxygeldanamycin hydrochloride (17-DMAG) to Tax-expressing ATL transformed cell lines, C8166 and MT4, induced significant degradation of Tax. 17-DMAG also facilitated growth arrest and cellular apoptosis to C8166 and MT4 and other ATL cell lines while this treatment has no apparent effects on normal PBLs. 17-DMAG also down-regulated Tax-mediated intracellular signals including activation of NF-κB, AP-1 or HTLV1-LTR in Tax-transfected HEK293 cells.

Oral administration of 17-DMAG to ATL-model mice xenografted with lymphomatous transgenic Lck-Tax cells or HTLV-1 producing tumor cells dramatically attenuated the aggressive infiltration into multiple organs, viral replication and improved survival periods. These observations identified 17-DMAG as a promising candidate for prevention of ATL progression.

